# Hearing impairment and incident disability and all-cause mortality in older British community-dwelling men

**DOI:** 10.1093/ageing/afw080

**Published:** 2016-09-08

**Authors:** Ann E. M. Liljas, S. Goya Wannamethee, Peter H. Whincup, Olia Papacosta, Kate Walters, Steve Iliffe, Lucy T. Lennon, Livia A. Carvalho, Sheena E. Ramsay

**Affiliations:** 1 Department of Primary Care and Population Health, University College London, London NW3 2PF, UK; 2 Population Health Research Centre, Division of Population Health Sciences and Education, St George's, University of London, London, UK; 3 Research Department of Epidemiology and Public Health, University College London, London, UK

**Keywords:** ageing, older adults, hearing impairment, disability, mortality

## Abstract

**Background and objective:** hearing impairment is common in older adults and has been implicated in the risk of disability and mortality. We examined the association between hearing impairment and risk of incident disability and all-cause mortality.

**Design and setting:** prospective cohort of community-dwelling older men aged 63–85 followed up for disability over 2 years and for all-cause mortality for 10 years in the British Regional Heart Study.

**Methods:** data were collected on self-reported hearing impairment including hearing aid use, and disability assessed as mobility limitations (problems walking/taking stairs), difficulties with activities of daily living (ADL) and instrumental ADL (IADL). Mortality data were obtained from the National Health Service register.

**Results:** among 3,981 men, 1,074 (27%) reported hearing impairment. Compared with men with no hearing impairment, men who could hear and used a hearing aid, and men who could not hear despite a hearing aid had increased risks of IADL difficulties (age-adjusted OR 1.86, 95% CI 1.29–2.70; OR 2.74, 95% CI 1.53–4.93, respectively). The associations remained after further adjustment for covariates including social class, lifestyle factors, co-morbidities and social engagement. Associations of hearing impairment with incident mobility limitations, incident ADL difficulties and all-cause mortality were attenuated on adjustment for covariates.

**Conclusion:** this study suggests that hearing problems in later life could increase the risk of having difficulties performing IADLs, which include more complex everyday tasks such as shopping and light housework. However, further studies are needed to determine the associations observed including the underlying pathways.

## Introduction

Hearing impairment increases with age and has been associated with chronic conditions including cardiovascular disease (CVD) and poor quality of life [1, 2]. Demographic changes mean that people are living longer with chronic diseases and associated physical limitations affecting independent living and overall well-being [3]. Disability in later life often occurs first as mobility limitations (for example, difficulties walking or climbing stairs) [4]. Other forms of disability refer to disablement in tasks essential to caring for oneself (basic activities of daily living [ADL], e.g. bathing, dressing) and more complex tasks that refer to living independently in the community (instrumental activities of daily living [IADL], e.g. shopping, telephoning) [4]. Earlier studies have shown an association between hearing impairment and mobility limitations [5] including increased risks of subsequent mobility limitations among older adults with hearing impairment [6, 7]. Previous research has also shown an association between hearing impairment and incident ADL deficits in hospital-based samples of older people [8, 9]. Hearing impairment has furthermore been associated with an increased risk of incident IADL; however, findings are inconsistent [6, 10–12]. In addition, it has been suggested that hearing impairment increases the risk of all-cause mortality [2], but some studies have shown no association after adjustment for demographic factors, physical functioning and cognition [12, 13].

It is important to understand the influence of hearing impairment on disability including activities of daily living to establish the impact of hearing impairment on functional independence in later life. Therefore, we investigated the association of self-reported hearing impairment with subsequent mobility limitations, ADL, IADL and all-cause mortality in a representative sample of older British men aged 63–85 followed up for 2 years for disability and 10 years for mortality. We also examined whether these associations were independent of age, social class, lifestyle factors and co-morbidities known to be associated with hearing impairment including CVD, hypertension and diabetes [1, 14].

## Methods

### Study design and participants

This study uses data from the British Regional Heart Study (BRHS), a prospective study in a socioeconomically and geographically representative sample of 7,735 middle-aged men drawn from 24 general practices representing all major British Regions [15]. The men were recruited in 1978–80 and have regularly been followed up since. For this study, baseline data on 3,981 men, then aged 63–85, were obtained through self-reported questionnaires in 2003. Ethical approval was obtained from relevant local research ethics committees.

### Hearing impairment

Questions on hearing impairment included ‘Do you use a hearing aid?’ and ‘Using a hearing aid if needed, is your hearing good enough to follow a TV programme at a volume others find acceptable?’ with answer options yes/no [16], and allowed for participants to be divided into four groups: could follow TV and used no hearing aid (could hear, no aid [no hearing impairment]) (reference group), could follow TV and used hearing aid (could hear, used aid), could not follow TV and did not use hearing aid (could not hear, no aid), and could not follow TV and used hearing aid (could not hear, used aid).

### Outcome measures

All men were followed up for mobility limitations and activities of daily living (ADL) and instrumental ADL (IADL) from 2003 to 2005 and for all-cause mortality from 2003 to 2013. Two questions asking whether they had problems taking the stairs and problems walking 400 yards with answer options yes/no were used to assess mobility limitation. Reporting problems with one or both was classified as having mobility limitations. ADL was classified as having some difficulty or in need of help undertaking one or more of the following activities: bathing, dressing, eating, getting in or out of bed or chair, toileting and/or walking across a room [17]. IADL was based on reporting some problem or in need of help undertaking cooking, shopping, using public transport, managing money and/or using the telephone [18]. Incidence was determined as having no previous mobility limitation, ADL and IADL, respectively. Mortality data were collected from the National Health Service register. Binary outcomes assessed in the current analyses were for incident mobility limitations, difficulties in ADL, IADL and all-cause mortality.

### Covariates

Covariates included socioeconomic and lifestyle factors including social class, social engagement, cigarette smoking, obesity and physical activity. Co-morbidity-related covariates included doctor-diagnosed CVD (coronary thrombosis, myocardial infarction, angina and/or stroke), hypertension and diabetes analysed dichotomously. Participants were divided into manual and non-manual social class based on the longest held occupation of subjects at study entry using the Registrar Generals' Social Class Classification. The men were grouped into non-smokers, ex-smokers and current smokers. Being obese was defined as having a body mass index (BMI) of 30 kg/m^2^ and over [19]. Physical activity scores were based on exercise type and frequency categorised as none, occasional, light, moderate, moderately vigorous and vigorous [20], where none or occasional activity was classified as being inactive. Other covariates included social engagement, doctor-diagnosed depression and difficulty keeping balance. Low social engagement was classified as doing three or fewer activities part of a 9-item social engagement scale on a weekly basis: voluntary work, go to the pub or a club, attend religious services, play cards or games, visit the cinema, restaurants or sports events, attend a class or course of study, and, sometimes go on day or overnight trips, and been on a holiday in the last year [21]. Depression and reporting not being able to keep balance were analysed dichotomously. Data on all covariates were collected at baseline (2003).

### Statistical analyses

Logistic regression was used to assess the associations of hearing impairment with incident mobility limitations and difficulties in ADLs and IADLs. Odds ratios (OR) with 95% confidence intervals (CI) were obtained using no hearing impairment (could hear, no aid) as reference group. Survival analysis was used to examine the association between hearing impairment and mortality, and Cox proportional hazards regression was used to calculate hazard ratios (HR) with 95% CIs. We also performed Cox regression using age as the time variable. The test confirmed that the proportionality hazards assumption was met. Participants who did not answer any of the hearing-related questions (*n* = 38) were excluded. Participants free from mobility limitations and difficulties in ADLs and IADLs at baseline were followed up for each of these types of disability. Models were adjusted for age, social class, lifestyle factors and co-morbidities. A Bonferroni correction was applied for multiple comparisons, and findings remained significant. All analyses were carried out using SAS version 9.3 software (SAS Institute, Inc., Cary, NC, USA).

## Results

In 2003, 3,981 men aged 63–85 completed the questionnaire (82% response rate). Of these, 3,108 men had no previous mobility limitations, 3,346 men had no previous ADL and 3,410 men had no previous IADL. At 2-year follow-up, there were 238 (8%) new cases of mobility limitations, 260 (8%) new cases of ADL and 207 (6%) new cases of IADL. All 3,981 men were also followed for all-cause mortality over 10 years during which 1,463 (37%) deaths occurred. Characteristics of participants by hearing impairment are shown in Table 1. Table 2 presents odds ratios (OR) with 95% CIs for incident mobility limitations, ADL and IADL for hearing impairment. Compared with men with no hearing impairment, men who could not hear and used a hearing aid had over a twofold greater risk of mobility limitations at 2-year follow-up (age-adjusted OR 2.24, 95% CI 1.29–3.89). The association remained after further adjustment for social class, lifestyle factors and co-morbidities (OR 1.89, 95% CI 1.04–3.41) but was attenuated upon adjustment for social engagement. Men who could not hear, irrespective of using hearing aid, had greater risks of developing problems performing ADL compared with men with no hearing impairment (OR 1.74, 95% CI 1.19–2.55; OR 2.01, 95% CI 1.16–3.46). The association was attenuated after further adjustment among men who used an aid but remained in those who could not hear and did not use hearing aid even after further adjustment for social engagement (OR 1.68, 95% CI 1.11–2.55). However, the association was attenuated after further adjustment for mobility limitations (OR 1.49, 95% CI 0.97–2.29). Compared with men with no hearing impairment, those who could hear and used a hearing aid and those who could not hear despite aid were more likely to develop IADL problems (OR 1.86, 95% CI 1.29–2.70; OR 2.74, 95% CI 1.53–4.93). These associations are of particular interest as they remained after further adjustment including social engagement (OR 2.00, 95% CI 1.34–2.99; OR 2.61, 95% CI 1.38–4.96) and also after further adjustment for mobility limitations, depression and poor balance (OR 2.03, 95% CI 1.35–3.07; OR 2.77, 95% CI 1.43–5.36). Further analyses of the associations between hearing impairment and individual components of IADL showed that men who could hear and used a hearing aid and men who could not hear despite aid were both more likely to experience problems, in particular undertaking shopping and light housework, even after further adjustment including social engagement. Men who could hear and used aid were also more likely to have problems using public transport. Only those who could not hear despite aid had increased risks of difficulty cooking (OR 2.03, 95% CI 1.05–3.94), but the association was attenuated after full adjustment (results not presented). Men who could hear and used aid and men who could not hear despite aid were more likely to have problems telephoning with over fourfold increased risks in men who could not hear despite aid (OR 4.53, 95% CI 2.25–9.10). The association remained in men who could not hear despite aid after further adjustment including social engagement (OR 4.29, 95% CI 2.02–9.13) and after further adjustment for mobility limitations, depression and poor balance (OR 4.29, 95% CI 2.00–9.18). The associations between hearing impairment and IADL were further analysed without the component of difficulty telephoning. Age-adjusted findings showed that men who could hear and used a hearing aid and men who could not hear despite an aid were more likely to develop difficulties in IADLs (results not presented). The association remained in men who could hear with aid only, after full adjustment including social engagement. None of the hearing impairment groups were associated with difficulties taking medications (results not presented).

**Table 1.** Percentages and numbers for socioeconomic and lifestyle characteristics, co-morbidities and mean age by hearing impairment in a cohort of British men aged 63–85 in 2003

% (*n*)	Overall	No hearing impairment	Hearing impairment groups			*P*-value
		Could hear, no aid	Could hear, used aid	Could not hear, no aid	Could not hear, used aid	
Totals	100 (3,981)	73 (2,851)	12 (482)	11 (424)	4 (168)	
Covariates						
Manual social class	51 (1,962)	48 (1,317)	53 (245)	63 (263)	60 (98)	<0.01
Current smokers	10 (389)	10 (284)	7 (33)	13 (54)	9 (15)	0.67
Ex-smokers	60 (2,385)	59 (1,681)	66 (314)	58 (244)	69 (115)	
Never smoked	30 (1,174)	31 (870)	27 (131)	29 (123)	22 (37)	
Physical inactivity	38 (1,430)	36 (971)	44 (196)	41 (157)	54 (87)	0.06
Obese	17 (639)	16 (445)	13 (61)	21 (85)	26 (42)	<0.01
CVD	27 (1,087)	26 (728)	32 (153)	26 (112)	43 (72)	<0.01
Hypertension	39 (1,547)	38 (1,092)	41 (196)	39 (165)	46 (78)	0.24
Diabetes	10 (393)	10 (281)	9 (45)	10 (41)	11 (19)	0.89
Age						
Mean age in years ± SD	72 (5.4)	72 (5.3)	75 (5.4)	72 (5.4)	74 (5.4)

**Table 2.** Odds ratios (OR) with 95% CIs for associations between incidence of mobility limitations, ADL and IADL and hearing impairment in British men aged 63–85 in 2003 followed up for 2 years to 2005

		No hearing impairment	Hearing impairment		
		Could hear, no aid	Could hear, used aid	Could not hear, no aid	Could not hear, used aid
Limitations in mobility	*n* (%)	150 (7)	39 (11)	23 (8)	17 (16)
Model 1	OR (95% CI)	1.00	1.40 (0.95–2.05)	1.16 (0.73–1.83)	2.24 (1.29–3.89)
Model 2		1.00	1.40 (0.92–2.12)	1.26 (0.78–2.03)	1.89 (1.04–3.41)
Model 2 + social engagement^a^		1.00	1.41 (0.93–2.14)	1.24 (0.77–2.01)	1.79 (0.98–3.27)
ADL		161 (7)	41 (10)	37 (11)	17 (15)
Model 1		1.00	1.30 (0.90–1.88)	1.74 (1.19–2.55)	2.01 (1.16–3.46)
Model 2		1.00	1.23 (0.82–1.84)	1.76 (1.16–2.66)	1.62 (0.90–2.94)
Model 2 + social engagement^a^		1.00	1.25 (0.83–1.87)	1.68 (1.11–2.55)	1.59 (0.87–2.88)
IADL		126 (5)	44 (11)	19 (6)	15 (15)
Model 1		1.00	1.86 (1.29–2.70)	1.09 (0.66–1.79)	2.74 (1.53–4.93)
Model 2		1.00	2.03 (1.36–3.01)	1.01 (0.59–1.75)	2.56 (1.35–4.86)
Model 2 + social engagement^a^		1.00	2.00 (1.34–2.99)	0.95 (0.54–1.67)	2.61 (1.38–4.96)
IADL components					
Shopping		73 (3)	31 (7)	17 (5)	13 (9)
Model 1		1.00	2.05 (1.32–3.20)	1.63 (0.95–2.80)	2.80 (1.50–5.23)
Model 2		1.00	1.96 (1.20–3.19)	1.56 (0.87–2.82)	2.39 (1.22–4.68)
Model 2 + social engagement^a^		1.00	2.01 (1.23–3.28)	1.46 (0.80–2.68)	2.30 (1.15–4.60)
Light housework		66 (2)	25 (6)	12 (3)	12 (8)
Model 1		1.00	1.93 (1.19–3.12)	1.24 (0.66–2.32)	3.08 (1.61–5.88)
Model 2		1.00	1.76 (1.05–2.95)	1.05 (0.54–2.05)	2.73 (1.39–5.34)
Model 2 + social engagement^a^		1.00	1.80 (1.07–3.04)	1.02 (0.52–2.00)	2.73 (1.39–5.38)
Telephoning		43 (2)	17 (4)	7 (2)	11 (8)
Model 1		1.00	1.85 (1.03–3.32)	1.10 (0.49–2.47)	4.53 (2.25–9.10)
Model 2		1.00	1.64 (0.88–3.04)	0.75 (0.29–1.93)	3.82 (1.80–8.09)
Model 2 + social engagement^a^		1.00	1.74 (0.93–3.24)	0.78 (0.30–2.03)	4.29 (2.02–9.13)
Managing money		59 (2)	16 (4)	10 (3)	14 (9)
Model 1		1.00	1.27 (0.71–2.25)	1.13 (0.57–2.23)	3.68 (1.99–6.82)
Model 2		1.00	1.29 (0.71–2.35)	0.97 (0.45–2.07)	3.49 (1.84–6.62)
Model 2 + social engagement^a^		1.00	1.32 (0.72–2.41)	0.95 (0.44–2.04)	3.68 (1.94–6.98)
Using public transport		75 (3)	33 (8)	13 (4)	7 (5)
Model 1		1.00	1.98 (1.28–3.06)	1.20 (0.66–2.20)	1.42 (0.64–3.19)
Model 2		1.00	1.97 (1.23–3.16)	1.16 (0.61–2.20)	1.33 (0.58–3.05)
Model 2 + social engagement^a^		1.00	1.93 (1.20–3.11)	1.13 (0.59–2.14)	1.36 (0.60–3.13)

Model 1: adjusted for age; Model 2: adjusted for age, social class, BMI, smoking, physical activity, CVD, hypertension and diabetes.

^a^Social engagement was defined as doing three or fewer of the following activities on a weekly basis: voluntary work, go to the pub or a club, attend religious services, play cards or games, visit the cinema, restaurants or sports events, attend a class or course of study, and, sometimes go on day or overnight trips, and been on a holiday in the last year.

Table 3 shows hazard ratios (HR) with 95% CIs for all-cause mortality associated with hearing impairment. Compared with men with no hearing impairment, those who could not hear and did not use a hearing aid had a significantly greater risk of all-cause mortality (HR 1.19, 95% CI 1.01–1.40) but the association was attenuated on further adjustments. No other hearing impairment group was associated with increased risk of all-cause mortality. These findings were confirmed when using age as the time scale.

**Table 3.** Hazard ratios (HR) with 95% CIs for associations between all-cause mortality and hearing impairment in British men aged 63–85 in 2003 followed up for 10 years to 2013

		No hearing impairment	Hearing impairment		
		Could hear, no aid	Could hear, used aid	Could not hear, no aid	Could not hear, used aid
	Rates/1,000 (*n*)	39 (974)	54 (216)	48 (169)	58 (76)
Model 1	HR (95% CI)	1.00	1.03 (0.88–1.19)	1.19 (1.01–1.40)	1.18 (0.93–1.49)
Model 2		1.00	1.01 (0.86–1.19)	1.12 (0.93–1.34)	1.14 (0.89–1.45)

Model 1: adjusted for age; Model 2: adjusted for age, social class, BMI, smoking, physical activity, CVD, hypertension and diabetes.

## Discussion

This study investigated the association of hearing impairment with incident disability (mobility limitations, ADL, IADL) and all-cause mortality in older British men. Our findings show that men with hearing impairment had greater risks in particular of disability affecting IADLs. The associations observed between hearing impairment and incident mobility limitations, incident ADL and all-cause mortality were attenuated on further adjustment for covariates.

The association between hearing impairment and mobility limitations was attenuated particularly on adjustment for social engagement. Communication problems due to hearing impairment may restrict social engagement [7]. Being socially engaged can motivate maintenance of physical functioning [22], reducing the risk of incident disability [23]. Only men who could not hear and did not use a hearing aid had greater risk of subsequent ADL deficits after adjustment including social engagement. However, the association was attenuated after further adjustment for mobility limitations. In contrast, men who could hear with an aid and men who could not hear despite an aid had increased risks of subsequent IADL difficulties and the associations remained after further adjustment. The associations also remained statistically significant after further adjustment for mobility limitations, depression and poor balance. This suggests that hearing impairment has a greater impact on IADLs which involve more complex tasks (such as shopping and light housework) than basic tasks including ADL and mobility limitations [24]. However, this finding should be interpreted with caution as the association between not being able to hear despite an aid and subsequent IADL was driven by difficulty telephoning. Also, the observed associations between hearing impairment and IADL could be explained by residual confounding due to unmeasured factors such as cognitive functioning, which is important for complex IADL tasks [13, 25]. The degradation hypothesis suggests that a decline in hearing impairment in older age increases the demands on cognitive functioning [8]. Previous research also suggests that family members may steer older relatives with poor physical and cognitive functions away from responsibilities and tasks such as IADLs [8]. Further, lack of consistent findings across the hearing impairment groups and incident IADL with no association observed in those ‘unable to hear, no aid’ suggests that this group may consist of a combination of men with a hearing problem who did not use a hearing aid due to, for instance, lack of access to health services and audiology assessments, reluctance to wear an aid, a perception that aids are unhelpful and men whose hearing problem is not improved by an aid. Finally, the association observed could be due to inflammation, which is related to both hearing impairment and disability [26, 27].

Men who could not hear and did not use hearing aid had greater risks of all-cause mortality compared with men with no hearing impairment. However, the association was attenuated after adjustment for social class, lifestyle factors and co-morbidities. This is consistent with earlier studies demonstrating no association after adjustment for potential confounders including social class and physical functioning [12, 13].

### Strengths and limitations

The major strengths of this study are that it was a large socioeconomically representative cohort with negligible loss to follow-up for disability and mortality [15]. In addition, the cohort was followed up for 2 years for disability and for 10 years for mortality, and the models were adjusted for several confounding variables.

Limitations include that hearing impairment was self-reported rather than objectively measured. However, the questions used have been validated against objective measures [16]. Furthermore, previous research has demonstrated comparable findings when investigating both self-reported (defined as ever had deafness or trouble hearing with one or both ears) and measured hearing impairment and 10-year mortality risk [12]. Further, the question on hearing aid use did not specify whether the participants have been offered a hearing aid and chosen not to use it or whether they do not have a hearing aid at all. Further, despite the large sample, the number of participants in each hearing impairment group with disability was small which might have reduced the statistical power of the study. Furthermore, hearing impairment was measured at baseline only, and no information on the primary cause of and change in hearing impairment were investigated. Finally, the study was in older men, predominantly of white British ethnic origin, and generalisation of findings to women and to other ethnic groups is limited.

## Conclusions and implications

In summary, our study shows that older men who could follow TV and used a hearing aid have greater risks of disability affecting IADLs, which are important for maintaining functional independence in later life. The inconsistent findings across the hearing impairment groups further suggest that it may not be hearing *per se* underlying the association. Future longitudinal studies are required to further assess the association between hearing impairment and incident disability, taking cognitive impairment and inflammation into account.

**Key points**

- Hearing problems in later life may increase the risk of having difficulties performing IADLs.
- Inconsistent findings across the hearing impairment groups suggest that something may be underlying the association with IADLs.
- The association of hearing impairment and all-cause mortality was attenuated on adjustment for covariates.

## Conflicts of interest

None declared.
